# Antarctic Epilithic Lichens as Niches for Black Meristematic Fungi

**DOI:** 10.3390/biology2020784

**Published:** 2013-05-17

**Authors:** Laura Selbmann, Martin Grube, Silvano Onofri, Daniela Isola, Laura Zucconi

**Affiliations:** 1 Department of Ecological and BiologicalSciences (DEB), University of Tuscia, Largo dell’Università snc, Viterbo 01100, Italy; E-Mails: onofri@unitus.it (S.O.); isola@unitus.it (D.I.); zucconi@unitus.it (L.Z.); 2 Institute of Plant Sciences, Karl-Franzens-University Graz, Holteigasse 6, A-8010 Graz, Austria; E-Mail: martin.grube@uni-graz.at

**Keywords:** black meristematic fungi, Dothideomycetes, Eurotiomycetes, lichen-associated fungi, phylogeny

## Abstract

Sixteen epilithic lichen samples (13 species), collected from seven locations in Northern and Southern Victoria Land in Antarctica, were investigated for the presence of black fungi. Thirteen fungal strains isolated were studied by both morphological and molecular methods. Nuclear ribosomal 18S gene sequences were used together with the most similar published and unpublished sequences of fungi from other sources, to reconstruct an ML tree. Most of the studied fungi could be grouped together with described or still unnamed rock-inhabiting species in lichen dominated Antarctic cryptoendolithic communities. At the edge of life, epilithic lichens withdraw inside the airspaces of rocks to find conditions still compatible with life; this study provides evidence, for the first time, that the same microbes associated to epilithic thalli also have the same fate and chose endolithic life. These results support the concept of lichens being complex symbiotic systems, which offer attractive and sheltered habitats for other microbes.

## 1. Introduction

Black meristematic fungi are known to be tolerant to extreme environmental conditions. The term black fungi embraces a polyphyletic group of fungi that share some phenotypic characters such as melanized cell walls and meristematic development, which seem to support survival and persistence in hostile environmental conditions. They are commonly isolated from environments that are almost devoid of other eukaryotic life-forms, including saltpans [1], acidic and contaminated sites [2,3,4], exposed rocks in dry and extremely hot or cold climates, ranging from hot deserts [5], the Mediterranean [6] to the Antarctic [7] and on monuments [8,9,10,11,12]. Owing to the stress pressure of the sites where they normally occur, black meristematic fungi are rarely found in complex microbial populations, rather they occur alone or in association with similar stress resistant organisms such as lichens [13,14] and cyanobacteria [15]. In the Antarctic, black meristematic fungi are recurrent members of endolithic microbial communities of ice free areas, including the lichen-dominated cryptoendolithic communities of the McMurdo Dry Valleys, one of the most inhospitable environments on Earth [16,17]. In these sites the limits for life are reached; since the conditions are too harsh to sustain epilithic settlement, mosses almost completely disappear and lichens grow protected in cracks and fissures or move inside the rocks, giving rise to well structured communities [16]. Together with lichens, other organisms can participate in these communities, in particular bacteria, cyanobacteria and non-lichenized fungi [18,19,20], but their biodiversity, their role and interactions are still scarcely investigated. Among these, the rock black fungi represent a peculiar group of colonizers [7,17].

Lichens are commonly described as a mutualistic symbiosis between fungi and “algae” (*Chlorophyta* or *Cyanobacteria*); however, recent studies revealed that they host a number of other microbes. Several culture-dependent and -independent studies have deepened our understanding of diverse populations of bacteria associated with lichens and their potential functional roles within the symbiosis [21,22,23,24,25]. Lichens also host numerous fungal species. The mycobiont is the dominant fungal species but other fungi may be present. These include lichenicolous fungi, which expresses symptoms [26,27] and endolichenic fungi, which grow without symptoms in the interior of lichens [28,29,30,31]. These studies have fueled the concept that, in addition to being symbiotic systems, where symbiotic partners may interact, lichens can also be considered miniature ecosystems [22,32]. However, despite a growing body of literature on organisms associated with lichens, we still have limited knowledge of the extent of eukaryotic diversity that may be associated with individual lichen thalli [31].

Lichens are commonly described as a mutualistic symbiosis between fungi and “algae” (Chlorophyta or ); however, recent studies revealed that they host a number of other microbes. Several culture-dependent and -independent studies have deepened our understanding of diverse populations of bacteria associated with lichens and their potential functional roles within the symbiosis [21,22,23,24,25]. Lichens also host numerous fungal species. The mycobiont is the dominant fungal species but other fungi may be present. These include lichenicolous fungi, which expresses symptoms [26,27] and endolichenic fungi, which grow without symptoms in the interior of lichens [28,29,30,31]. These studies have fueled the concept that, in addition to being symbiotic systems, where symbiotic partners may interact, lichens can also be considered miniature ecosystems [22,32]. However, despite a growing body of literature on organisms associated with lichens, we still have limited knowledge of the extent of eukaryotic diversity that may be associated with individual lichen thalli [31].

In this study we focused on extremotolerant black fungi associated with cold-loving lichens from the Antarctic, including the endemic species *Lecanora fuscobrunnea* Dodge & Baker. Antarctic lichens are still an unexplored niche for these organisms and we aimed to compare the black fungi diversity among different lichen species distributed in diverse ecosystems.

## 2. Experimental Section

Sampling sites and lichen identification: lichen thalli were collected using a sterile chisel and preserved in sterile plastic bags at −20 °C until processed for isolation of associated fungi. Lichens were identified using the key by Castello [33]. All data concerning the sampling sites and the identifications are reported in Table 1.

**Table 1:** Lichen species analyzed collection data and fungal strains isolated.

Lichen species	Location	Coordinates	Sampling date	Fungal strains (CCFEE)
*Acarospora* sp.	Ford Peak, NVL	75°41'26.3''S 160°26'25.3''E	28/01/2004	-
*Acarospora flavocordia* Castello & Nimis	Kay Island, NVL	75°04'13.7''S 165°19'02.0''E	30/01/2004	5324
*Buellia frigida* Darb.	Inexpressible Island, NVL	75°52'23.2''S 163°42'16.5''E	17/01/2004	-
*Lecanora fuscobrunnea* Dodge & Baker	Edmonson Point, NVL	74°19'43.7''S 165°08'00.7''E	29/01/2004	5320 *
*Lecanora fuscobrunnea* Dodge & Baker	Convoy Range, Terra SVL	76°54'33.0''S 160°50'00.0''E	25/01/2004	5303
*Lecanora* sp.	Inexpressible Island, NVL	75°52'23.2''S 163°42'16.5''E	17/01/2004	5319 *, 5323
*Lecidea* sp.	Starr Nunatak, NVL	75°53'55.7''S 162°35'31.3''E	15/02/2004	5318
*Lecidea* sp.	Starr Nunatak, Terra Vittoria del Nord	75°53'55.7''S 162°35'31.3''E	15/02/2004	5326
*Lecidea* *cancriformis* Dodge & Baker	Widowmaker Pass, NVL	74°55'23.5''S 162°24'17.0''E	12/02/2004	5321 **
*Rhizocarpon* sp.	Vegetation Island, NVL	74°47'05.2''S 163°38'40.3''E	16/01/2004	5312
*Umbilicaria aprina* Nyl.	Kay Island, NVL	75°04'13.7''S 165°19'02.0''E	30/01/2004	-
*Umbilicaria decussata* (Vill.) Zahlbr.	Kay Island, NVL	75°04'13.7''S 165°19'02.0''E	02/02/2004	-
*Umbilicaria decussata* (Vill.) Zahlbr.	Vegetation Island, NVL	74°47'05.2''S 163°38'40.3''E	16/01/2004	5317
*Usnea antarctica* Du Rietz	Kay Island, NVL	75°04'13.7''S 165°19'02.0''E	30/01/2004	-
*Usnea antarctica* Du Rietz	Vegetation Island, NVL	74°47'05.2''S 163°38'40.3''E	16/01/2004	5313 *
*Xanthoria elegans* (Link) th. Fr.	Kay Island, NVL	75°04'13.7''S 165°19'02.0''E	30/01/2004	5314, 5322

CCFEE—Culture Collection of fungi From Extreme Environments; NVL—Northern Victoria Land; SVL—Southern Victoria Land. Identified strains: * *Elasticomyces elasticus*; ** *Friedmanniomyces endolithicus*.

Isolation: in order to remove any potential contaminant before isolation, lichens were treated with H_2_O_2_ (8%) for 5 min; H_2_O_2_ was removed by washing with distilled sterile water for 5 min. The solution was filtered using 500 µm porosity filters. All fragments were collected and seeded on MEA (Malt Extract Agar, Oxoid, Ltd. Basingstoke, Hampshire, UK) in Petri Dishes and incubated at 5 °C and 15 °C. Plates were inspected weekly and as soon as new black colonies appeared they were transferred on fresh agar slant. Pure cultures were deposited in the CCFEE (Culture Collection of Fungi from Extreme Environments, DEB, Università degli Studi della Tuscia, Viterbo, Italy).

Morphology and temperature preferences: hyphal maturation was studied using light microscope. Slide cultures were seeded onto MEA, incubated for 10 w and mounted in lactic acid. Temperature preferences were performed in triplicate on MEA, in Petri dishes in the range 0–30 °C ± 1, with 5 °C intervals. Colony diameters were recorded monthly.

Molecular analysis: DNA was extracted from 6-months-old mycelium grown on MEA at 10 °C, using Nucleospin Plant kit (Macherey-Nagel, Düren, Germany) following the protocol optimized for fungi. Target gene for our analysis was the nuclear ribosomal 18S and ITS genes. PCR reactions were performed using BioMix (BioLine, Luckenwalde, Germany) and primers NS1-NS24 and ITS1-ITS4 to amplify 18S and ITS respectively [34]. Reaction mixtures were prepared by adding 5 pmol of each primer and 40 ng of template DNA in a final volume of 25 μL. For amplification, a MyCycler™ Thermal Cycler (Bio-Rad Laboratories, Munich, Germany) was used. The protocol used for amplification of the nuclear ribosomal 18S was as follows: 3 min at 95 °C for a first denaturation step, a denaturation step at 95 °C for 45 s, annealing at 52 °C for 30 s. Cycles were repeated 35 times, with a last extension at 72 °C for 5 min. ITS portion was amplified as previously described [7]. Products were purified using Nucleospin Extract kit (Macherey-Nagel, Düren, Germany). Sequencing reactions were performed according to the dideoxynucleotide method using the TF BigDye Terminator 1,1 RR kit (Applied Biosystems). Fragments were analyzed by Macrogen Inc. (Seoul, Korea). Sequence assembly was done using the software ChromasPro (version 1.32, Technelysium, Conor McCarthy School of Health Science, Griffith University, Southport, Queensland, Australia).

The alignment based on nuclear ribosomal 18S included 79 sequences of strains belonging to the class *Dothideomycetes* and *Eurotiomycetes* in the public domain chosen on the base of the Blastn results. Additional sequences of black fungi deposited in the database of the CCFEE (Culture Collection of Fungi from Extreme Environments, Università degli Studi della Tuscia, Viterbo, Italy) were analyzed (Table 2). Sequences were aligned iteratively with ClustalX [35], exported in Mega5 [36] for a manual improvement. The best-fit substitution model and Maximum Likelihood phylogenetic tree reconstruction was performed as previously described [17]. The robustness of the phylogenetic inference was estimated using the bootstrap method [37] with 1000 pseudoreplicates.

**Table 2:** List of strains and sequences analyzed.

Species	Strains no.	Source	Location	SSU
*Acidomyces acidophilum*	C2	acid mine drainage	CA, USA	AY374300
*Acidomyces acidophilum*	A3-7	acid mine drainage	CA, USA	AY374299
*Acidomyces acidophilum*	B1	acid mine drainage	CA, USA	AY374298
*Aureobasidium pullulans*	28v1	-	-	AY137505
*Aureobasidium pullulans*	30v4	-	-	AY137507
*Botryosphaeria ribis*	CBS 121.26	*Ribes rubrum*	-	U42477
*Botryosphaeria ribis*	CBS 115475	*Ribes*	-	DQ678000
*Capnobotryella renispora*	CBS 214.90	*Abies*	Japan	EF137360
*Capnobotryella renispora*	CBS 215.90	*Sphagnum*	Japan	AY220613
*Capnobotryella renispora*	CBS 572.89	Roof tile	Sweeden	AY220614
*Capnobotryella renispora*	UAMH 9870	*Sphagnum*	-	AY220611
*Capronia coronata*	CBS 617.96	Decorticated wood	New Zealand	AJ232939
*Capronia semiimmersa*	CBS 840.69	Decaying timber	Finland	AY554291
*Catenulostroma abietis*	CBS 459.93	*Abies*	Germany	DQ678040
*Cladophialophora carrionii*	CBS 260.83	Skin lesion	-	AY554285
*Cladophialophora* sp.	CBS 985.96	Brain	USA	AJ232953
*Coccodinium bartschii*	UME30232	-	-	U77668
*Coniosporium* sp.	MA 4597	Marble	Turkey	AJ972863
*Cyphellophora laciniata*	MUCL 9569	-	-	AY342010
*Cryomyces antarcticus*	CCFEE 514	Rock	Antarctica	GU250319
*Cryomyces antarcticus*	CCFEE 515	Rock	Antarctica	GU250320
*Cryomyces antarcticus*	CBS 116301T; CCFEE 534	Sandstone	Antarctica	DQ028269
*Cryomyces minteri*	CBS 116302; CCFEE 5187	Sandstone	Antarctica	DQ028270
*Discosphaerina fagi*	CBS 171.93	*Populus* leaf	UK	AY016342
***Elasticomyces elasticus***	**CBS 122538; CCFEE 5313**	**Lichen**	**Antarctica**	**FJ415474**
***Elasticomyces elasticus***	**CBS 122539; CCFEE 5319**	**Lichen**	**Antarctica**	**GU250332**
***Elasticomyces elasticus***	**CBS 122540; CCFEE 5320**	**Lichen**	**Antarctica**	**GU250333**
*Elsinoe centrolobii*	CBS 222.50	*Centrolobium robustum*	Brazil	DQ678041
*Exophiala salmonis*	CBS 157.67	*Salmo clarkii*	Canada	JN856020
*Exophiala salmonis*	AFTOL-ID 671	-	-	EF413608
*Friedmanniomyces endolithicus*	CCFEE 670	Rock	Antarctica	GU250322
*Friedmanniomyces endolithicus*	CCFEE 5208	Rock	Antarctica	Unpublished
***Friedmanniomyces endolithicus***	**CCFEE 5321**	**Lichen**	**Antarctic**	**Unpublished**
*Fonsecaea pedrosoi*	CBS 272.37	-	-	AY554290
*Guignardia mangiferae*	IFO 33119	*Rhododendron pulchrum*	-	AB041247
*Guignardia mangiferae*	CBS 226.77	*Paphiopedilum callosum*	-	AB041248
*Guignardia mangiferae*	CBS 398.80	Orchid	-	AB041249
*Hobsonia santessonii*	-	-	-	AF289658
*Hortaea werneckii*	dH10921	Marble	-	Y18700
*Hortaea werneckii*	CBS 107.67	human *Tinea nigra*	-	Y18693
*Knufia chersonesos*	CBS 600.93; dH16058	Marble	Greece	Y18702
*Knufia chersonesos*	CBS 726.95	Marble	Italy	Unpublished
*Knufia perforans*	CBS 885.95	Marble	Delos, Greece	Y11714
*Knufia perforans*	CBS 665.80	Marble	Delos, Greece	Y11712
*Mycocalicium victoriae*	CBS 109863	Soil	Italy	Unpublished
*Myriangium duriaei*	CBS 260.36	*Chrysomphalus*	Argentina	NG_013129
*Pseudotaeniolina globosa*	CBS 109889	Rock	Italy	GU214576
*Saxomyces alpinus*	CCFEE 5466	Rock	Alps, Italy	GU250350
*Saxomyces alpinus*	CCFEE 5469	Rock	Alps, Italy	KC315860
*Saxomyces alpinus*	CCFEE 5470	Rock	Alps, Italy	KC315861
*Saxomyces penninicus*	CCFEE 5495	Rock	Alps, Italy	KC315864
*Recurvomyces mirabilis*	CBS 119434; CCFEE 5264	Rock	Antarctica	GU250329
*Rhinocladiella atrovirens*	CBS 688.76	*Pinus*	Australia	AJ232937
Rock black fungus	CCFEE 451	Rock	Antarctic	GU250314
Rock black fungus	CCFEE 457	Rock	Antarctic	GU250317
Rock black fungus	CCFEE 507	Rock	Antarctic	Unpublished
Rock black fungus	CCFEE 5176	Rock	Antarctic	GU250325
Rock black fungus	CCFEE 5177	Rock	Antarctic	Unpublished
Rock black fungus	CCFEE 5205	Rock	Antarctic	GU250327
Rock black fungus	CCFEE 5207	Rock	Antarctic	Unpublished
Rock black fungus	CCFEE 5267	Rock	Antarctic	Unpublished
Rock black fungus	CCFEE 5284	Rock	Antarctic	GU250330
Rock black fungus	CCFEE 5303	Rock	Antarctic	GU250331
Rock black fungus	CCFEE 5329	Rock	Antarctic	Unpublished
*Teratosphaeria microspora*	CBS 101951; STE-U 1960	Leaf	South Africa	EU167572
*Teratosphaeria molleriana*	CPC 1214	*Eucalyptus globulus*	Portugal	GU214606
*Teratosphaeria molleriana*	CPC 4577	*Eucalyptus*	Australia	GU214582
*Teratosphaeria molleriana*	CPC 10397	*Eucalyptus globulus*	Spain	GU214607
*Teratosphaeria nubilosa*	CPC 933	*Eucalyptus nitens*	South Africa	GU214608
*Teratosphaeria nubilosa*	CPC 937	*Eucalyptus globulus*	Australia	GU214609
**Unknown black fungus**	**CCFEE 5304**	**Lichen**	**Antarctic**	**Unpublished**
**Unknown black fungus**	**CCFEE 5312**	**Lichen**	**Antarctic**	**Unpublished**
**Unknown black fungus**	**CCFEE 5314**	**Lichen**	**Antarctic**	**Unpublished**
**Unknown black fungus**	**CCFEE 5317**	**Lichen**	**Antarctic**	**Unpublished**
**Unknown black fungus**	**CCFEE 5318**	**Lichen**	**Antarctic**	**Unpublished**
**Unknown black fungus**	**CCFEE 5322**	**Lichen**	**Antarctic**	**GU250334**
**Unknown black fungus**	**CCFEE 5323**	**Lichen**	**Antarctic**	**Unpublished**
**Unknown black fungus**	**CCFEE 5324**	**Lichen**	**Antarctic**	**Unpublished**
**Unknown black fungus**	**CCFEE 5326**	**Lichen**	**Antarctic**	**Unpublished**

AFTOL—Assembling Fungal Tree Of Life project; CBS—Centraalbureau voor Schimmelcultures; CCFEE—Culture Collection of Fungi From Extreme Environments; CPC—Culture collection of P Crous, housed at the CBS; dH—de Hoog private collection housed at the CBS; IFO—Institute for Fermentation Culture Collection, Japan; MUCL—Belgian Co-ordinated Collections of micro-organisms; STE-U—University of Stellenbosch fungal culture collection, Stellenbosch, South Africa; UAMH—The University of Alberta Microfungus Collection and Herbarium, Edmonton, AB, Canada; UME—Herbarium Department of Ecology and Environmental Sciences (EMG) University of Umeå, Sweden. Strains isolated in this study are reported in bold.

## 3. Results and Discussion

**Figure 1:**
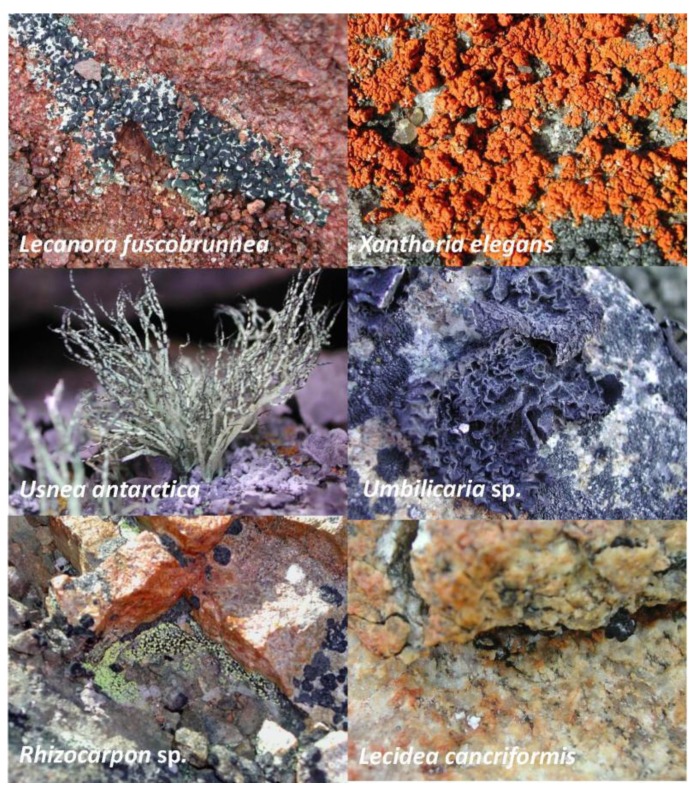
Some of the lichen thalli examined for black fungi.

**Figure 2:**
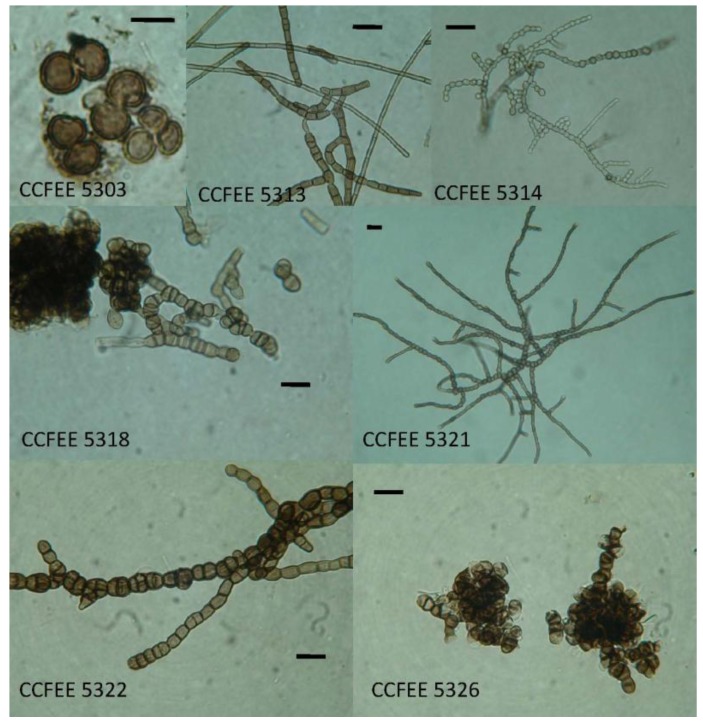
Some of the black fungi isolated from the lichens examined. This is a selection made based on morphological and phylogenetic characteristics.

Data concerning lichen sample (Figure 1), collection sites and black fungi isolated are reported in Table 1. The epilithic vegetation is rather rare in the Dry Valleys, it is therefore not surprising that only one lichen sample, *Lecanora fuscobrunnea*, out of 16 studied, was collected in Southern Victoria Land. Black fungi (Figure 2) were recovered from 11 out of 16 lichens examined.

Temperature relations are given in Table 3. All the strains tested were able to grow at 0 °C and none of the strains grew at 30 °C. Strains 5303, 5314, 5317, 5321, 5324, 5326 had their optimal growth temperature at 15 °C and did not show any growth above that temperature. All these strains can therefore be classified as psychrophilic, as defined for yeasts and other eukaryotic microorganisms [38]. Strain 5323, with optimal temperature and upper limit for growth at 20 °C, also may be defined as psychrophilic. Peculiar temperature relations, highlighting a more eurythermic behavior, were observed for strains 5313, 5319, 5320 with optimum at 15 °C but 25 °C as upper limit, too high for a true psychrophilic fungus. A similar profile was observed for strain CCFEE 5318 but with an optimal temperature at 20 °C.

**Table 3:** Temperature relations.

	Temperature (°C)						
Isolates	0	5	10	15	20	25	30
**CCFEE 5303**	6.8 ± 1.8	4.3 ± 2.5	11.3 ± 0.4	**13.8 ± 1.8**	-	-	-
**CCFEE 5312**	6.9 ± 1.3	7.8 ± 1.1	11.8 ± 0.4	**18.2 ± 1.9**	5.2 ± 1.6	-	-
**CCFEE 5313**	16.4 ± 0.8	12.9 ± 0.6	25.8 ± 0.4	**31.9 ± 3.7**	30.8 ± 1.1	19.5 ± 3.5	-
**CCFEE 5314**	4.4 ± 0.6	5.2 ± 1.6	7 ± 0	**31.9 ± 3.7**	-	-	-
**CCFEE 5317**	4.8 ± 0.4	3.3 ± 0.4	8 ± 1.4	**12 ± 0**	-	-	-
**CCFEE 5318**	7.3 ± 0.4	4.1 ± 1.8	13.3 ± 0.4	13 ± 0.4	**13.5 ± 0.7**	11.3 ± 3.2	-
**CCFEE 5319**	15.5 ± 0.7	11.9 ± 0.1	22 ± 0	**30 ± 0.7**	24 ± 0.7	11.8 ± 1.1	-
**CCFEE 5320**	14 ± 0.7	10.8 ± 1.1	23 ± 1.4	**27.7 ± 0.9**	22.5 ± 0.7	16.8 ± 1.8	-
**CCFEE 5321**	3.5 ± 0.7	4.7 ± 0.9	8.8 ± 0	**10.3 ± 1.8**	-	-	-
**CCFEE 5322**	10.3 ± 1.8	9.8 ± 1.1	14 ± 1.4	**18.4 ± 2.3**	4 ± 0	-	-
**CCFEE 5323**	7 ± 0.7	4.7 ± 0.9	7 ± 2.1	18 ± 0.7	**20.5 ± 0.7**	-	-
**CCFEE 5324**	3.5 ± 0.7	4.1 ± 1.8	5.3 ± 0.4	**8.8 ± 0**	-	-	-
**CCFEE 5326**	2.5 ± 0	3.5 ± 0.7	4.1 ± 1.8	**7 ± 0.7**	-	-	-

Growth are reported as diameter of the colonies (mm) after 3 months of incubation. Highest growth values are reported in bold.

**Figure 3:**
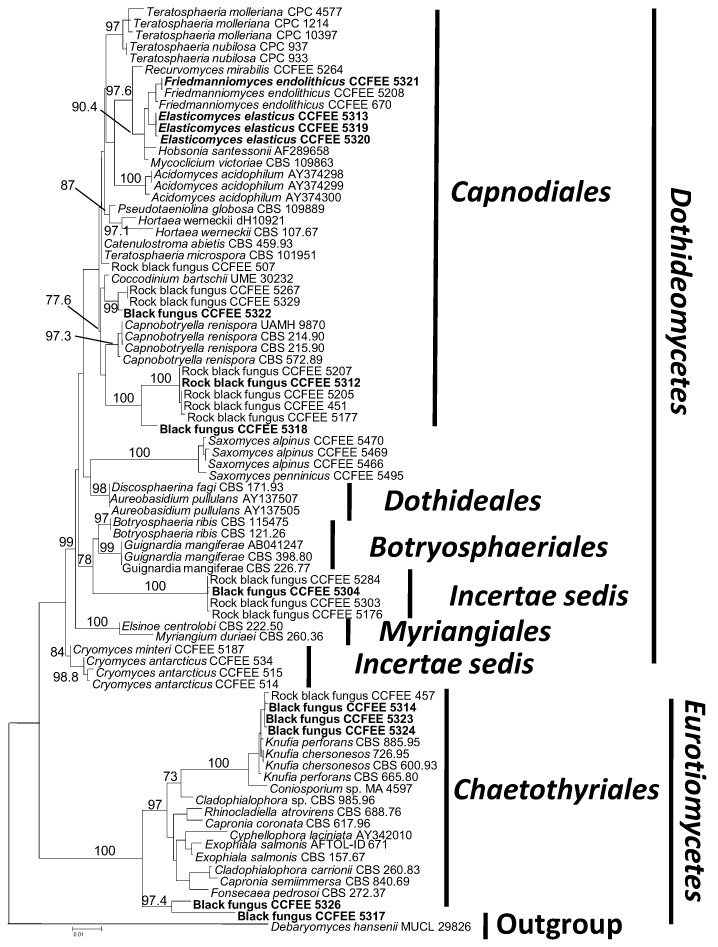
SSU ML tree indicating the phylogenetic position of the black meristematic fungi isolated from lichens (reported in bold in the tree). The strains reported as Rock black fungus are still unidentified rock fungi deposited in the Culture Collection of Fungi From Extreme Environments. Bootstrap values are the results of 1,000 pseudoreplicates. Values below 70 are not shown.

Most of the ITS sequences obtained showed too low identities in the GenBank and were not used for the phylogenetic inference. Figure 3 shows the ML phylogenetic tree, generated using a GTR+IG model, which was selected using the Akaike’s information criterion with a Maximum likelihood approach. The alignment was based on 79 nuclear ribosomal 18S gene sequences and 1707 positions, including gaps, belonging to strains of both plant pathogenic and rock fungi, some of which were still unidentified. The tree includes two classes within the Ascomycota: Dothideomycetes (Orders Capnodiales, Dothideales, Myriangiales and Botryosphaeriales) and Eurotiomycetes (Order Chaetothyriales). The tree was rooted with *Debaryomyces hansenii* MUCL 29826.

The backbone remains uncertain, but orders in the class Dothideomycetes, are resolved although the 18S gene only was compared. The tree is in agreement with the most recent phylogenetic analyses with the Order Botryosphaeriales separated from Capnodiales [39,40]. Two sister clades are segregated in the Order Chaetothyriales: the group comprising most of the human opportunists of the family Herpotrichiellaceae as *Cladophialophora carrionii* (Trejos) de Hoog, Kwon-Chung & McGinnis, and the clade composed of mostly rock fungi, including the genus *Knufia* [41].

Seven of the strains here studied were grouped in the order Capnodiales placed in lineages purely constituted of fungi from rocks. Strains CCFEE 5312 and 5318 are included in a wide clade of rock fungi [40]; here only a selection of strains from the Antarctic is included, but the clade comprises rock fungi from the Mediterranean and Alps too, as well as the melanised micro-filamentous lichen *Cystocoleus ebeneus* (Dillwyn) Thwaites [42]. The strain CCFEE 5322 groups with rock black fungi exclusively from the Antarctic. The remaining strains in the Capnodiales belong to the rock fungal species *Elasticomyces elasticus* Zucconi & Selbmann and *Friedmanniomyces endolithicus* Onofri [43], the last one exclusively from the Antarctic continent [7,17]. The strain CCFEE 5304 as included in a well separated and supported clade of rock fungi from Antarctic rocks collected both in Northern and Southern Victoria land colonized with endolithic communities. This group remains without a clear assignment at any known fungal order. The remaining five strains were in the order Chaetothyriales (class Eurotiomycetes). Strains CCFEE 5326 and 5317 grouped together in a separated position with high bootstrap value and do not show clear relations with any described or undescribed species in the tree the ITS sequences were only 88% similar with the closest deposited in GenBank: this is not uncommon for black fungi from locations where genetic and geographic isolation, coupled with environmental pressure, promoted adaptive radiation [7]. Yet, their long branches indicate that these strains are distantly related to each other. Strains CCFEE 5314, 5323 and 5324 cluster with a rock Antarctic fungus, CCFEE 457, isolated from sandstone collected in the Dry Valleys; this group of Antarctic black fungi is sister of a clade represented by the recently formalized genus *Knufia* [41], including species mainly isolated from monuments.

All the strains examined here are not related to groups that contain known lichenicolous species. Rather, they show strict phylogenetic relations with fungi occurring on and in Antarctic rocks. Likewise, the rock black fungi included in this study were found to belong to two classes of Ascomycota: Dothideomycetes and Eurotiomycetes, in this last case specifically in the order Chaetothyriales (Figure 3). Dothideomycetous rock black fungi prefer natural, non-contaminated environments, while chaetotyriomycetous rock black fungi are recurrent particularly in areas influenced by human activities, rich in pollutants [44] probably as consequence of their ability to metabolize aromatic compounds [45].

Lichens can host a wide range of associated fungi with varied ecologies, specificities, and biological traits [26]. Some fast-growing lichenicolous species (e.g., *Athelia*, *Marchandiomyces*), with often low host specificity, can rapidly eradicate lichen vegetation, whereas many others grow slowly without expressing any or only local pathogenic symptoms on their specific hosts, apparently as a long-term result of evolutionary adaptation [46]. These lichenicolous fungi are not found to express their phenotypes without their hosts. Some groups of black fungi have also been observed to colonize a wide range of lichens, as lichenicolous fungi. Some species in the genus *Lichenothelia*, a cosmopolitan genus of rock-inhabiting melanised fungi in the superclass dothideomyceta [47] have been found in association with algae or with lichen thalli, where they produce fertile structures with asci and ascospores. However, species reported in this study were not related to *Lichenothelia* nor with any of the groups comprising known lichenicolous fungi. Moreover, they do not produce visible symptoms on thalli. Several melanized fungi were isolated from lichens from Armenia and the Alps with obscure discolourations [14] belonging to the genera *Mycosphaerella*, *Rhinocladiella*, *Capnobotryella* (class Dothideomycetes) and *Coniosporium*, in this last case related to *Knufia perforans* (Sterflinger) Tsuneda (class Eurotiomycetes). The strains CCFEE 5314, 5323, 5324 isolated during this study may be related to this last species, but the above mentioned isolates were not included in our tree since the SSU sequences are not available. Comparing the ITS sequences of our isolates we found that they were 10% distant from the sequences FJ265756 (*Coniosporium* sp. h6) and FJ265754 (*Coniosporium* sp. c-SH-2009a) isolated form *Caloplaca saxicola* (Hoffm.) Nordin and *Protoparmeliopsis muralis* (Scherb.) M. Choisy respectively, both from Armenia.

Association of black fungi with primary producers could be interpreted from a nutrition-ecological point of view. Oligotrophy is important adaptation for life on rocks and these fungi may often rely only on the sparse, airborne nutrients available, as pollutants in urban environments [45]. In natural environments, with low anthropogenic impact and scarce nutrient availability, they could more conveniently obtain nutrients, by living in association with lichens and other microbial primary producers, such as algae and cyanobacteria, which reside in the endolithic microbial communities of the highest mountain peaks and Antarctica [7,17,48].

Interestingly, some rock-inhabiting species were previously observed to develop lichen-like structures in axenic cultures with phototrophic algae [49,50]. This ability to develop symbiotic interactions with unicellular free-living algae might have allowed some rock-inhabiting fungal lineages to evolve lichenisation and a common link between rock-inhabiting meristematic and lichen-forming lifestyles of ascomycetous fungi has been recently hypothesized [47]. Some studies suggest that some rock-inhabiting fungi constitute early diverging lineages for lichenized fungal groups as Verrucariales and Arthoniomycetes [51].

## 4. Conclusions

This study represents the first contribution regarding black fungi associated with lichen thalli in the Antarctic. All strains isolated were either closely related or conspecific with black fungi previously found associated with Antarctic endolithic microbial communities. These are mostly cryptoendolithic lichen dominated communities, on the borderlines of what life can tolerate [16]. Even from cosmopolitan lichen species, we isolated endemic Antarctic fungi as *F. endolithicus*. Data obtained in this study give new insights into the biology of lichens: they are particularly well adapted to survive in extreme conditions and the ability to vary microbial communities associated according with the location may give further advantage in adaptation and survival of the whole community. 

It is still unclear whether or not black fungi may supply benefits to epilithic lichens as well. It was suggested previously [7] that black fungi may play a role in in hydration or protection of photobionts by dissipating excessive sunlight. Cryptoendolitic lichens are melanized fungi that form a black barrier just above the photobionts stratification [14] making this a plausible scenario. The presence of black fungi may therefore play a crucial role to allow survival in these highly stressful conditions. 

Apparently, at the cold edge of life, lichens, together with their associated microbes could find a solution to survive inside the rock by taking advantage of the suite of traits from each microbial partner in order to improve stress resistance and allow the whole community to survive in new conditions.
